# Effects of Different Nanocarbon Materials on the Properties of Al/MoO_3_/NCM Thermite Prepared by Electrostatic Spinning

**DOI:** 10.3390/nano12040635

**Published:** 2022-02-14

**Authors:** Mi Zhang, Hui Ren, Qingzhong Cui, Hanjian Li, Yongjin Chen

**Affiliations:** 1 State Key Laboratory of Explosion Science and Technology, Beijing Institute of Technology, Beijing 100081, China; 15903461635@163.com (M.Z.); renhui@bit.edu.cn (H.R.); chenyongjin122@163.com (Y.C.); 2 Beijing Research Institute of Mechanical & Electrical Technology, Beijing 100081, China; lihanjian@yahoo.com

**Keywords:** Al/MoO_3_/NCM, electrostatic spinning, thermal conductivity, energy performance, combustion performance

## Abstract

In order to improve thermal conductivity, energy performance, and combustion performance of the aluminum-containing thermite, nanocarbon materials were added to thermite. Aluminum/molybdenum and trioxide/nanocarbon materials (Al/MoO_3_/NCM) were fabricated by electrostatic spinning technology. The Al and MoO_3_ particles of the nAl/MoO_3_/NCM thermite are much smaller than nitrocellulose (NC); thus, the two components can be better attached to NC fibers. Results on thermal conductivity demonstrated that the addition of NCM can improve the thermal conductivity of Al/MoO_3_, and the addition of reduced graphene oxide (RGO) has a more significant impact on thermal conductivity. Energy performance analysis results indicated that the energy performance of Al/MoO_3_/NCM thermite spinning is the best when the value of combustion oxygen equivalent ratio (Φ) is 0.90–1.00. The combustion performance results show that the addition of NCM can significantly increase the combustion rate of thermites, and the addition of RGO improves its combustion rate the most, followed by carbon nanotubes (CNT) and nanoflake graphite (NFG) being the lowest. By changing the shape of the Al/MoO_3_/NCM charge and the internal composition of the charge, the sensitivity of the agent can be adjusted, and the matching performance and use performance of the electric igniter can be improved.

## 1. Introduction

Thermite is a mixture of metal fuel and metal oxide, and it can undergo a violent oxidation–reduction reaction under external energy stimulation. Thermite has been widely used in the energy of incendiary agents, ignition powders, high-energy explosives, and solid rocket propellants [1,2]. Due to the large size of raw materials, the mass transfer rate between the reactants of traditional thermite is slow, resulting in a slow energy release rate and incomplete extortion of work potential. Nanothermite uses nanotechnology to achieve full mixing and interface contact between metal fuel and oxide particles at the nanoscale [3,4,5]. Due to its excellent combustion and energy properties, such as high energy density, adjustable energy release rate, ignition sensitivity and reaction speed, and non-toxic reaction products, nanothermite has attracted widespread attention in the past decade.

Nanocarbon materials (NCMs) have a catalytic effect on the reaction of energetic systems. Adding a handful of NCM to energetic material can improve the overall performance of the energetic material [6]. Graphite has excellent electrical conductivity, thermal conductivity, lubricity, etc. Adding a small amount of graphite to energetic materials, such as explosives and propellants, can increase dispersion properties, prevent static electricity, change the burning rate, and reduce sensitivity [7,8]. Carbon nanotubes are functionalized or combined with other substances and then introduced into the energetic material system, which can improve ignition, energy release, and other properties of the agent [9,10,11,12]. Determination of the effective properties of CNT-reinforced composite is a highly complicated issue, for there are many factors that could affect the overall response. These include the interfacial load transfer condition, surface functionalization to improve load transfer and CNT dispersion, and CNT waviness and agglomeration, among others [13,14,15]. The formation of CNT agglomeration and an imperfect interface can seriously reduce the elastic stiffness and yield strength of the nanocomposite [13]. Hassanzadeh-Aghdam found that among three dispersion patterns (agglomerated, uniformly dispersed, and aligned states), the lowest creep modulus belongs to the agglomerated pattern while the alignment of graphene nanoplatelet results in the highest value [16]. Reduced graphene oxide is a derivative of graphene prepared by chemical methods [17,18]. It has a graphene-like hexagonal lattice structure with a small number of functional groups and certain defects remaining in the plane. It decomposes to produce gas at about 200 °C. Reduced graphene oxide has good thermal conductivity performance, which is expected to improve the disadvantages of high ignition threshold and low gas production.

Electrospinning is a general technology for preparing micro–nano-composite materials. It has the advantages of simple operation, continuity, and high efficiency [19,20,21]. The prepared micro–nano-composite fiber has the characteristics of high specific surface area and high porosity, which can effectively avoid the problems of agglomeration and uneven dispersion of single micro–nano particles [22,23,24]. At the same time, it also has the performance of multiple components; thus, it becomes an ideal carrier for sub-micron energetic particles. In the electrospinning process, the fiber diameter can be controlled and adjusted in the range of nanometers to microns by adjusting the parameters, such as working voltage, feed rate, polymer solution concentration, conductivity, and so on [25,26]. At the same time, nanofiber composite materials with different surface structures and functions can be obtained by changing the composition of the polymer matrix solution, thereby meeting different functional requirements of fiber materials in different fields.

In this paper, Al/MoO_3_/NCM composites were prepared by the electrospinning technique. Nanocarbon materials were introduced into Al/MoO_3_ as an important additive. The morphology and structure were analyzed by SEM, EDS, and XRD. The thermite by adding NCM shows excellent thermal conductivity and combustion performance than Al/MoO_3_.

## 2. Materials and Methods

### 2.1. Materials

Al particles were purchased from Aladdin Industrial Co., Ltd. (Shanghai, China) (nano-Al (nAl, the D_50_ is 50 nm); micron-Al (mAl, the D_50_ is 25 um); submicron-Al (sub-mAl, the D_50_ is 800 nm)); molybdenum trioxide (MoO_3_, D_50_ is 40 nm, 20 um, and 630 nm) was obtained from Beijing Tongguang Fine Chemical Co., Ltd. (Beijing, China); hexane (purity of 99.5%) and absolute ethyl alcohol (purity of 99.5%) was purchased from Tianjin Fuyu Fine Chemical Co., Ltd. (Tianjin, China); reduced graphene oxide (RGO) was provided by Chinese Academy of Sciences Chengdu Organic Chemistry Co., Ltd. (Chengdu, China); carbon nanotube (CNT) was obtained from Nanjing Xianfeng Nanomaterials Technology Co., Ltd. (Nanjing, China); nanoflake graphite (NFG) was provided by Beijing Deke Island Gold Technology Co., Ltd. (Beijing, China); collodion (NC, 4–8 wt.%) was purchased from Shanghai Aladdin Bio-Chem Technology Co., Ltd. (Shanghai, China); and perfluorinated polyether (PFPE) was obtained from Solvay Plastics Co., Ltd. (Shanghai, China).

### 2.2. Characterizations

A scanning electron microscope (SEM) was used to observe the surface micromorphology of the prepared thermite spinning and its components (SEM, S4800, Hitachi Ltd., Tokyo, Japan), and an X-ray energy spectrometer (EDS, EMAX, Horiba Ltd., Tokyo, Japan) was used to analyze the types and contents of microcomponent elements of the samples. The X-ray diffraction spectrum of the sample was obtained by X-ray diffraction (XRD, D8-Advance X-ray diffractometer, Bruker Co. Ltd., Beijing, China); the X-ray source selected for the test is Cu-K_α rays with a wavelength of 0.154 nm; scanning angle range of 5–80°; scanning rate of 4°·min^−1^ and scanning step length of 0.02°; and the test temperature is 25 °C. The thermal conductivity of the thermite spinning was measured by the heat flow thermal conductivity meter (DRL-III-C, Xiangtan Xiangyi Instrument Co. Ltd., Xiangtan, China). The sample needs to be pressed into a disc with a certain thickness. The diameter of the sample is 30.0 mm, and the thickness is about 1 mm. The temperature of the cold electrode plate of the tester is 25 °C, the temperature of the hot electrode plate is 50 °C, and the test pressure is 800 KPa. A high-speed camera was used to record the burning state of the thermite spinning in PMMA tubes of different inner diameters after ignition, and the burning rate was calculated according to the correspondence between the burning process and the time (high-speed camera, FASTCAM APX RS, Photron Co. Ltd., Tokyo, Japan; capacitor discharge initiator, ALG-CN2, Nanjing University of Science and Technology, Nanjing; PMMA, φ4~7 mm, Tianchang Ruici plexiglass Co. Ltd., Tianchang, China).

### 2.3. Preparations

Accurately weigh a certain amount of Al and mix it with a small amount of PFPE in a beaker in an ultrasonic environment in order to ensure that the surface of the Al powder is coated by PFPE. Then, add a certain amount of MoO_3_ and NCM in proportion, and disperse the mixture powder in 20 mL n-hexane under ultrasonic vibration to form a uniform suspension. Place the beaker in a ventilated and high-power ultrasonic environment and heat it at 50 °C until the n-hexane is completely volatilized. Collect the compound powder in the beaker and place the compound powder in a blast drying oven at 60 °C for 3 h. In a 50 mL beaker, mix the dried composite powder with a certain volume of collodion solution (5% V) to form a suspension solution. Place the beaker in a magnetic stirrer for about 5 minutes for preliminary mixing. Finally, place the beaker in an ultrasonic cell pulverizer and shake for about 15 minutes so that the components of the thermite are uniformly dispersed in the collodion solution. Since the solvent in collodion is very volatile, an appropriate amount of absolute ethanol can be added to adjust the concentration of the solution during experimental operations. The MoO_3_ particles and Al particles not coated with PFPE are mixed in proportion, and collodion is added to prepare a spinning precursor solution for Al/MoO_3_ spinning preparation. In the configuration process of the above spinning suspension solution, according to the principle of minimum free energy, the addition amount of NCM is determined to be 4%, and the formula ratio of the thermite agent is Al:MoO_3_:NC = 18%:48%:30%.

**Figure 1:**
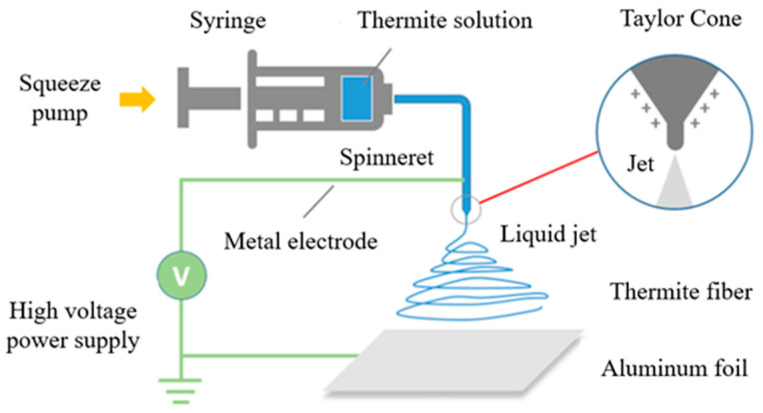
Diagram of electrostatic spinning.

The electrospinning Al/MoO_3_/NCM was prepared by electrostatic spinning. The internal diameter of the needle was 0.8 mm. The liquid precursors were inhaled into the injector and squeezed at a feed rate of 4.5 mL/h. The needle was connected to a high-voltage power supply. The voltage was fixed at 18 kV to form a taylor cone. Al foil was used as the receiving substrate and placed 6 cm away from the tip of the needle. The electrospinning process is shown in Figure 1.

## 3. Results and Discussion

### 3.1. Morpology and Structure

**Figure 2:**
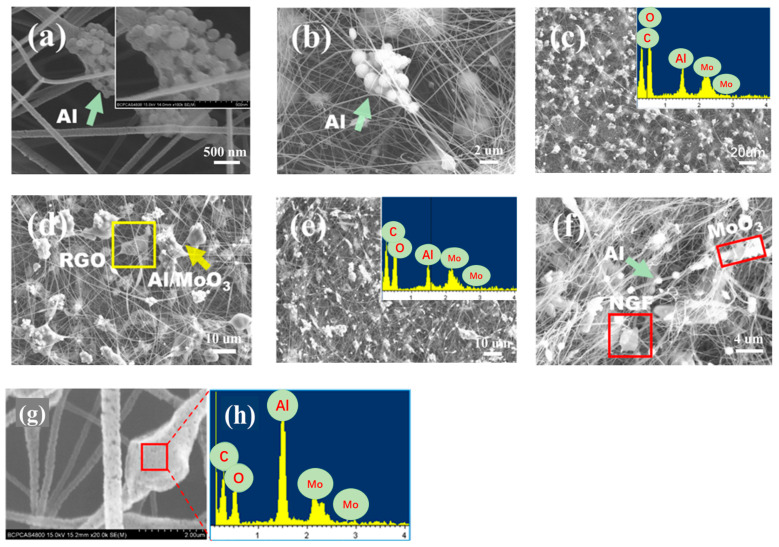
Microscopic morphology and element distribution map of Al/MoO_3_/NCM spinning (**a**), and (**b**) is the SEM images of nAl/MoO_3_ and mAl/MoO_3_, in which Al is not coated with PFPE; (**c**) is the SEM diagram and microregion element distribution diagram of sub-mAl/MoO_3_/RGO spinning, in which (**d**) is the high-power SEM diagram of (**c**); (**e**) is the SEM diagram and microregion element distribution diagram of sub-mAl/MoO_3_/NFG spinning, in which (**f**) is the high-power SEM diagram of (**e**); (**g**) is the SEM diagram of nAl/MoO_3_/RGO; (**h**) is the microregion elements distribution diagram of (**g**).

The microscopic morphology and microdomain element distributions of the Al/MoO_3_/NCM composite prepared by electrospinning are shown in Figure 2. nAl and mAl without PFPE coating will settle and aggregate due to different densities when they are slowly extruded in the syringe in Figure 2a,b and separate from MoO_3_ particles. Al particles showed obvious agglomeration; thus, they could not be effectively mixed with MoO_3_ particles. This phenomenon of uneven mixing and component separation will directly affect the performance of the prepared thermite. Al and MoO_3_ particles coated with PFPE are not easy to settle and separate in the thermite suspension solution, and NCM has a small density and can be stably suspended in the mixed solution; thus, uniform Al/MoO_3_/NCM spinning can be obtained by electrostatic spinning. Although the particles of the micron and sub-micron Al/MoO_3_/NCM thermite cannot be attached to the NC fiber, the components can be effectively dispersed and uniformly mixed in Figure 2c,f, which can be proved from the corresponding microzone element distribution map.

Al and MoO_3_ particles of the nAl/MoO_3_/NCM thermite are both nanosized, and their particle size is much smaller than NC; thus, the two components can be better attached to NC fibers. However, the NCM used in the experiment is submicron or even micron in at least one dimension; thus, even if the Al/MoO_3_/NCM thermite particles are used for spinning, the bead-like structure are formed or mixed particles are directly free from spinning. The micromorphology and element distribution diagrams of nAl/MoO_3_/RGO spinning are shown in Figure 2g,h. The spinning surface presents a concave–convex and intermittent structure. This is due to the fact that nAl and nMoO_3_ are filled in NC, but the solvent in NC evaporates and shrinks. Microarea element analysis was performed on a similar beaded area on Al/MoO_3_/RGO spinning, and the element type distribution map was obtained. It can be observed from Figure 2h that there are four elements of C, O, Mo, and Al in the structures, which proves the existence of thermite components.

**Figure 3:**
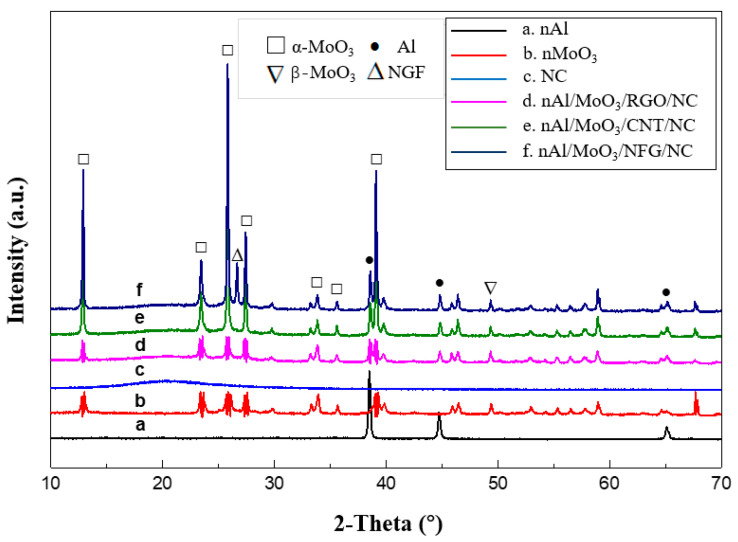
XRD spectra of Al/MoO_3_/NCM spinning and raw materials.

The XRD spectra of nAl/MoO_3_/NCM spinning and raw materials are shown in Figure 3. Figure 3a is the XRD spectrum of nAl particles. There are three obvious diffraction peaks in the curve. The diffraction peaks at the diffraction angles of 38.47°, 44.78°, and 65.09° corresponded to (1 1 1), (2 0 0), and (2 2 0) crystal planes of Al crystals (JCPDS Card No. 04–0787) for the face-centered cubic system. Figure 3b is the XRD spectrum of nMoO_3_ particles. The diffraction angles 2θ in the spectrum are 12.98°, 23.58°, 25.92°, 27.48°, 33.98°, and 39.20°, corresponding to (0 2 0), (1 1 0), (0 4 0), (0 2 1), (1 1 1), and (1 5 0) crystal planes of orthorhombic system α-MoO_3_ (JCPDS Card No. 21–0506), and the diffraction peak at 2θ of 49.53° is the (0 6 1) crystal plane of β-MoO_3_ for the monoclinic system (JCPDS Card No. 76–1003). The XRD spectrum of pure NC shows that there is a wide amorphous dispersion peak at 2θ of about 20°, indicating that NC exists in an amorphous form. Figure 3d–f are the XRD spectra of nano Al/MoO_3_/RGO, Al/MoO_3_/CNT, and Al/MoO_3_/NFG spinning, respectively. The amorphous dispersion peaks of NC, crystal sharp peaks of Al, and MoO_3_ particles appeared in three spectra. The diffraction peak at 26.66°in Figure 3f is the (0 0 2) crystal plane of graphite (JCPDS Card No. 41–1487).

### 3.2. Thermal Conductivity

Thermal conductivity is an important thermal physical performance parameter of thermite, which is directly related to the heat transfer of thermite, the difficulty of ignition, and the ability to ignite. It can be used to qualitatively characterize the heat transfer effect of the thermite after being thermally stimulated, and it can also indirectly evaluate the ease of ignition and thermal safety of the pharmaceutical system. In order to compare the thermal conductivity of aluminum-containing thermite after adding carbon materials, the heat flow method was used to test the thermal resistance of nAl/MoO_3_ and nAl/MoO_3_/NCM, and the thermal conductivity and thermal resistance of the material were calculated by Formulas (1) and (2) [27]:

(1) $$K_{T}=A\cdot \left[\frac{T_{h}-T_{c}}{x}\right]\cdot {\left[\frac{\Delta Q}{\Delta t}\right]}^{-1}$$

(2) $$K_{T}=A\cdot \left[\frac{T_{h}-T_{c}}{x}\right]\cdot {\left[\frac{\Delta Q}{\Delta t}\right]}^{-1}$$

where *K_T_* is the thermal conductivity of the sample at temperature *T*; *A* is the area of the test plate; *T_h_* and *T_c_* are the temperatures of the hot and cold plates, respectively; x is the distance between the two plates; Δ*Q*/Δ*t* is the amount of heat transferred by the sample in unit time, which is heat transfer rate; *R_T_* is the thermal resistance of the sample at temperature *T*; and *d* is the thickness of the measured material.

**Figure 4:**
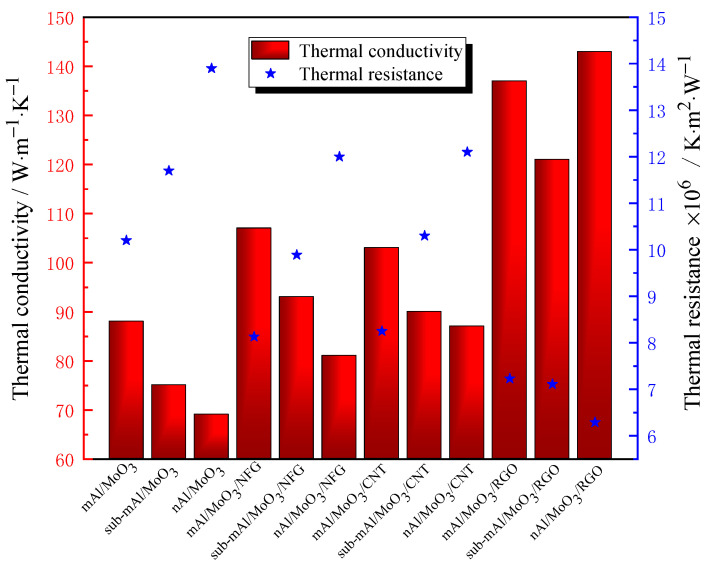
The histogram of thermal resistance and thermal conductivity for thermite.

The thermal conductivity test results of the thermite are shown in Table 1, and the histogram of thermal resistance and thermal conductivity for thermite is exhibited in Figure 4. It can be observed from Figure 4 that the thermal conductivities of Al/MoO_3_ thermite spinning of micron, submicron, and nanosize grades are similar but gradually decrease. Compared with Al/MoO_3_ thermite particles, the thermal conductivity of the three particle sizes of Al/MoO_3_ thermite spinning significantly decreased by about 24.8%, 27.2%, and 28.1%, respectively (the thermal conductivity of mAl/MoO_3_, sub-mAl/MoO_3_, and nAl/MoO_3_ thermite particles are 117 W·m^−1^·K^−1^, 103 W·m^−1^·K^−1^, and 96 W·m^−1^·K^−1^, respectively). The thermal conductivity of nAl/MoO_3_ thermite spinning is reduced the most. The thermal conductivity of Al/MoO_3_ thermite spinning after NC coating or bonding is not only affected by the thermal conductivity of NC but also related to changes in internal structure. Due to the existence of gaps between the raw material component of micro–nano Al/MoO_3_ thermite spinning, overall thermal conductivity is reduced. In addition, the NC with poor thermal conductivity is coated on the surface of the particles, which reduces the thermal conductivity of the system, and the spinning structure has a larger porosity. Therefore, the combined effect of these factors makes the thermal performance of Al/MoO_3_ thermite spinning significantly reduced than compared to Al/MoO_3_ thermite particles. For nAl/MoO_3_ thermite spinning, the nanoparticles have a larger specific surface area. After the spinning process, more NC components are coated on the surface of nanoparticles, which hinders heat transfer at the interface of the nanoparticles. Compared with the spinning of micron and submicron particles, the spinning of nanoparticles has a larger porosity, and the thermal conductivity of the system has a negative correlation with the void fraction. Hence, the thermal conductivity of nAl/MoO_3_ thermite spinning reduced most obviously.

Nanocarbon materials have good thermal conductivity, and adding to the thermite can improve the thermal conductivity of the thermite system. The thermal conductivity of the three particle sizes of Al/MoO_3_ thermite spinning has been significantly improved after adding NFG and CNT. Thermal conductivity is improved the most by sub-mAl/MoO_3_/NFG and nAl/MoO_3_/CNT, and it increased by 24.0% and 26.1%, respectively. The above two nanocarbon materials have similar effects on the thermal conductivity of micron and submicron Al/MoO_3_ thermite spinning, but CNT has a more significant increase in the thermal conductivity of nAl/MoO_3_, resulting in the thermal conductivity of nAl/MoO_3_/CNT being higher than nAl/MoO_3_/NFG. After adding RGO, the thermal conductivity of the three particle sizes of Al/MoO_3_ thermite spinning has been improved more significantly. In particular, the thermal conductivity of nAl/MoO_3_/RGO is higher than sub-mAl/MoO_3_/RGO and mAl/MoO_3_/RGO. Thermal conductivity increased by 55.7%, 61.3%, and 107.2% in the order of micron, submicron, and nanolevels compared with Al/MoO_3_, respectively. The addition of RGO can significantly improve the thermal conductivity of Al/MoO_3_ thermite spinning, especially for nAl/MoO_3_.

### 3.3. Energy Performance

**Figure 5:**
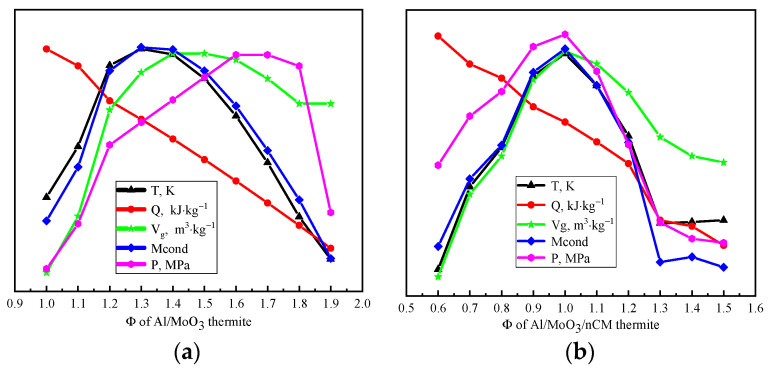
(**a**) Energy parameters of Al/MoO_3_ thermite, (**b**) Energy parameters of Al/MoO_3_/NCM thermite.

According to the principle of minimum free energy, combustion temperature, combustion heat, gas-phase product volume, solid residue volume, and constant volume gas product pressure of Al/MoO_3_/NCM thermite spinning with different fuel–oxygen equivalent ratios at the reaction equilibrium were calculated. The thermite is 1 kg, the environmental pressure during the reaction is 0.1 MPa, the system is an adiabatic system, and system iteration accuracy is 10^−6^. The calculation results are shown in Table 2 and Table 3. Figure 5 shows the energy parameter change curve of Al/MoO_3_/NCM thermite spinning with different combustion oxygen equivalent ratios, Φ.

Combustion temperature, combustion heat, gas-phase product volume, solid residue volume, and constant volume gas product pressure reflect the comprehensive effect of each microreaction in the Al/MoO_3_/NCM thermite spinning system. It can be observed from Figure 5a that the combustion temperature and solid residue amount of Al/MoO_3_ thermite spinning show a trend of increasing and then decreasing with the gradual increase in Φ value. The values of combustion temperature and solid residue amount are the highest when the value of Φ is 1.30~1.40. The change curve of the gas production volume and the constant volume gas product pressure increased and then gradually flattened, while combustion heat still shows a unilateral downward trend, indicating that the increase in the value of Φ is not conducive to the release of combustion heat. The amount of reducing agent continues to increase while the amount of oxidant continues to decrease, which is not conducive to the full progress of the reaction. In order to measure the combined effect of energy parameters, Φ = 1.30–1.40 was selected as the optimal fuel–oxygen equivalent ratio for the energy performance of Al/MoO_3_ thermite spinning. Figure 5b shows that the combustion heat of Al/MoO_3_/NCM thermite spinning still shows a downward trend. The values of the other energy parameters are all at the top of the parabola when the value of Φ is 0.90~1.00. Other parameters reach the maximum except for the heat of combustion at this time. Therefore, the energy performance of Al/MoO_3_/NCM thermite spinning is the best when the value of Φ is 0.90–1.00.

### 3.4. Combustion Performance

**Figure 6:**
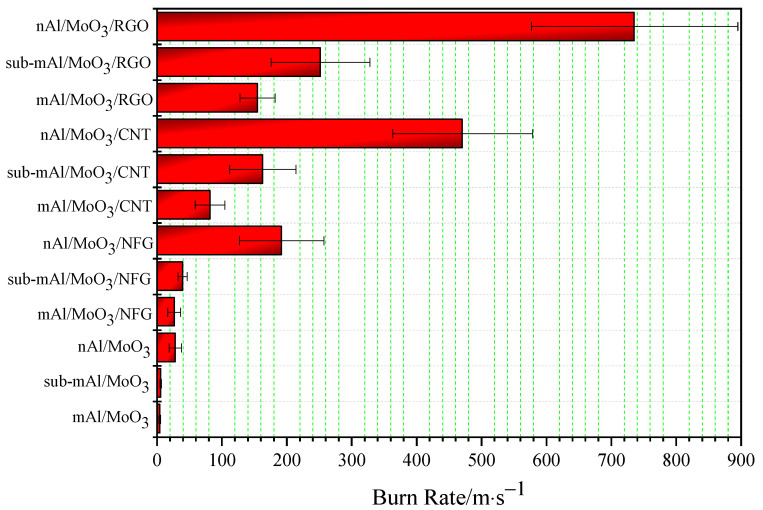
The histogram of linear burning rate.

Before testing the burning rate of micro–nano thermite spinning, it is necessary to peel off the spinning filament attached to the aluminum foil with tweezers and then roll it into a cylindrical shape. Then, micro–nano thermites were packed into a PMMA tube with a certain inner diameter and the packing density was kept at 1.73 g·cm^−3^. Use the electric ignition head to ignite the thermite spinning from one end of the PMMA tube under the action of the capacitor discharge detonator. The combustion process of thermite was recorded by a high-speed camera, and the linear burning rate of the thermite film was calculated according to the correspondence between the burning process and time. Each thermite sample was tested three times in parallel, and the standard deviation was calculated. The results are shown in Table 4, and the histogram of the linear burning rate is exhibited in Figure 6.

The burning rate of Al/MoO_3_ thermite spinning of a micron, submicron, and nanometer is low. Among them, the burning rate of mAl/MoO_3_ thermite spinning is the lowest, and nAl/MoO_3_ has the highest burning rate; that is, the smaller the particle size is, the higher the burning rate will be. The highest burning rate is about seven times the lowest burning rate. This change law is the opposite of thermal conductivity. Due to the existence of gaps between the raw material particles of thermite spinning, overall thermal conductivity is reduced. However, the particle size of nanoparticles is much smaller than microparticles, and nanoparticles have a higher specific surface area. The pore volume generated by the accumulation of nanoparticles is greater than microparticles. Therefore, when the particle size of the raw materials used in Al/MoO_3_ thermite spinning is smaller, the increase in its specific surface area will increase the contact area between the oxidant and reducing agent particles in the thermite, which will promote the rapid acceleration of the thermite reaction. At the same time, the reduction in particle size will increase the void fraction of the system such that a small part of the high-temperature thermite particle steam produced by the reaction acts on thermite spinning at the back end of the reaction, which promotes the decomposition reaction of NC and penetrates the thermite, thus accelerating heat transfer and heat convection in the system. The simultaneous action of the heat transfer and heat convection can significantly increase the burning rate of Al/MoO_3_ spinning.

The burning rate of Al/MoO_3_ thermite spinning for micron, submicron, and nanometer significantly increased to about 6.3, 7.1, and 6.8 times when NFG was added, but the standard deviation of the burning rate for nAl/MoO_3_/NFG is relatively large due to unstable combustion and increased burning rate; that is, the burning process has a large fluctuation. The burning rate of Al/MoO_3_ increased to about 19.3, 29.3, and 16.8 times by adding CNT. After adding RGO, the burning rate is more significantly improved than adding NFG and CNT. Compared with the three particle sizes of Al/MoO_3_ thermite spinning, the combustion rate of Al/MoO_3_/RGO increased to about 36.6, 45.3, and 26.2 times in the order of a micron, submicron, and nanometer. It can be observed that the addition of RGO can significantly improve the burning rate of Al/MoO_3_ spinning, especially the burning rate of nAl/MoO_3_. In short, the addition of nanocarbon materials can significantly increase the combustion rate of the thermite. The addition of RGO improves its combustion rate the most, followed by CNT, and NFG is the lowest. In the combustion process of thermite spinning, binder NC reacts preferentially. Under the simultaneous action of external energy stimulation, it promotes the rapid response of thermite particles in spinning. The nanocarbon material undergoes oxidation reaction under the action of hot thermite particles to generate high temperature and pressure gas products and penetrates the unreacted porous thermite spinning condensed phase interface to promote heat convection and mass transfer processes in thermite spinning. In turn, it initiates the decomposition of NC and the rapid redox reactions between the thermite particles in frontend thermite spinning. Therefore, the addition of nanocarbon materials can significantly increase the burning speed of thermite spinning.

**Figure 7:**
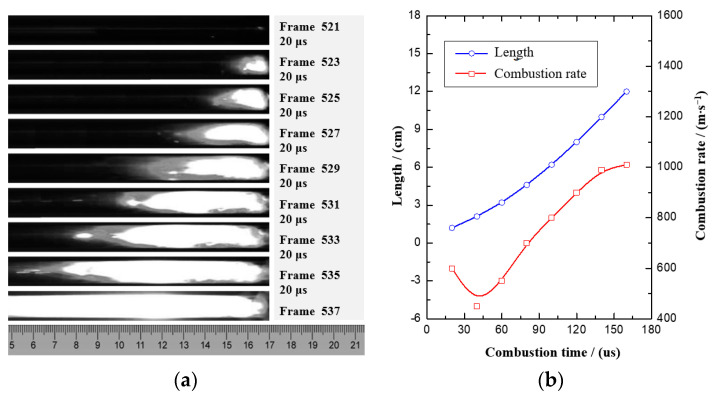
The graph of the combustion process (**a**) and combustion rate (**b**) of nAl/MoO_3_/RGO.

It can be observed from the above analysis that nAl/MoO_3_/RGO has the highest burning rate. To further explore the microscopic change law of the burning rate of thermite spinning with RGO, the combustion process and burning rate change law of nAl/MoO_3_/RGO thermite spinning were specifically analyzed. Figure 7 shows the combustion process and the graph of the combustion rate of nAl/MoO_3_/RGO. The average burning rate of nAl/MoO_3_/RGO thermite spinning under the weak restriction of the PMMA tube is 736 m·s^−1^, and the highest burning rate is 1100 m·s^−1^. Certain fluctuations at the front of the combustion flame are mainly due to the higher porosity of the spinning structure and the higher burning rate. It takes about 160 μs for the combustion wave of nAl/MoO_3_/RGO thermite spinning to propagate 120 mm in the PMMA tube. At the beginning of combustion, the combustion wave has an initial step of about 20 μs, and then it undergoes a rapid rise phase of about 100 μs. The combustion rate stabilizes after the final combustion wave travels about 100 mm and maintains the highest combustion rate. The change in the burning rate indicates that nAl/MoO_3_/RGO thermite spinning can quickly reach a combustion equilibrium state after starting to burn for about 140 μs under external energy stimulation.

### 3.5. Electrothermal Ignition Characteristics

**Figure 8:**
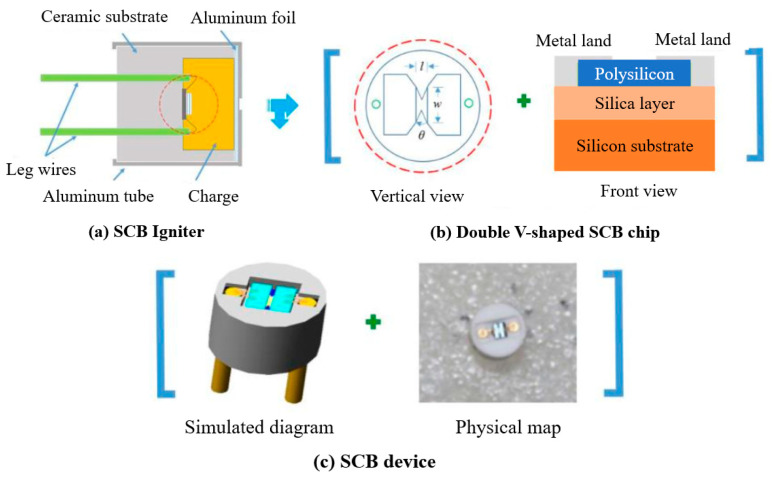
Structure diagram of semiconductor bridge firing parts: (**a**) SCB Igniter, (**b**) Double V-shaped SCB chip, (**c**) SCB device.

The semiconductor bridge pyrotechnic device has high safety, fast response characteristics, low ignition energy, high reliability, and high ignition consistency. It is considered to be a revolutionary intelligent ignition device that can be combined with microelectronic circuits and digital logic. The circuit is compatible and has been successfully used in microelectromechanical systems (MEMS). In this experiment, the ignition characteristics of Al/MoO_3_/RGO were evaluated by semiconductor bridge ignition parts. The characteristics of the two semiconductor bridge transducer models (D1 and D2) used in the experiment are the length of the bridge area (l), the width of the bridge area (w), the number of V-shaped angles (θ), the area of the ignition area (A), and resistance (Ω) and other parameter descriptions, as shown in Table 5. In addition, the thickness of the bridge region of these two types of semiconductor bridges is 2 μm, and the substrate is made of ceramic material. The diameter of D1 is 4.4 mm and that of D2 is 6.0 mm. Al/MoO_3_/RGO thermite is uniformly packed at the bottom of the aluminum shell by pressing, and then the SCB transducer is placed. The aluminum shell, medicine, and SCB device are packaged into micro–nano thermite-charged SCB-fired parts. The pressing pressure is 7 MPa, the charge volume is 50 mg, and the charge density is 2.63 g·cm^−3^. The structure of the semiconductor bridge ignition component is shown in Figure 8.

Under the excitation of capacitor discharge, the critical ignition voltage of the semiconductor bridge ignition element containing the micro–nano thermite fiber and the film charge was tested. In order to compare the electrothermal pyrophoric performance of micro–nano thermite with the commonly used pyrophoric agents of electrothermal transducers, two types of SCB transducers were charged with neutral lead styphnate (N-LS) and combined charge (N-LS/LA) of N-LS and lead azide (LA). The results are shown in Table 6. The ignition of the ignition element assembled by the D1 semiconductor bridge transducer is that the high temperature and pressure metal vapor generated by the explosion of the semiconductor bridge penetrate the agent and can cause the agent to ignite. This mechanism is defined as the electric explosion fire mechanism. The ignition of the ignition parts assembled from the D2 semiconductor bridge converter is caused by the semiconductor bridge heating agent, which increases the agent temperature to its ignition point and fire. This mechanism is defined as an electrothermal ignition mechanism.

It can be observed from Table 6 that the critical ignition voltage of the D2 semiconductor bridge transducer is much larger than D1; that is, D2 requires higher energy stimulation than D1 under the excitation of capacitor discharge. The reason is that the latter has a fire zone area much larger than the former, which can disperse energy on the entire planet and requires a larger voltage to cause it to fuse or explode. For the discharge capacitor excitation and electrothermal ignition mechanism, the critical ignition voltages of the semiconductor bridge ignition components D2-nAl/MoO_3_/RGO (fiber) and D2- nAl/MoO_3_/RGO (membrane) are higher than the semiconductor bridge ignition components containing N-LS/LA charge, indicating that the required fire stimulation energy of the nAl/MoO_3_/RGO fiber and nAl/MoO_3_/RGO membrane is higher than that of N-LS/LA charge, and the required fire stimulation energy of nAl/MoO_3_/RGO fiber is slightly higher than that of N-LS/LA charge. The critical ignition voltage of the three types of semiconductor bridge ignition element is lower than the critical voltage for the explosion or fusing of the semiconductor bridge transducer; that is, the semiconductor bridge transducer element will ignite without fuse. It fully shows that the three types of semiconductor bridge ignition components belong to the electrothermal ignition mechanism. By conducting the critical ignition voltage test, it can be observed that the ignition sensitivity of the agent can be adjusted by changing the form and composition of the Al/MoO_3_/RGO charge and making it compatible with N-LS and N- LS/LA, which can improve its matching performance and use performance on the semiconductor bridge ignition component.

**Figure 9:**
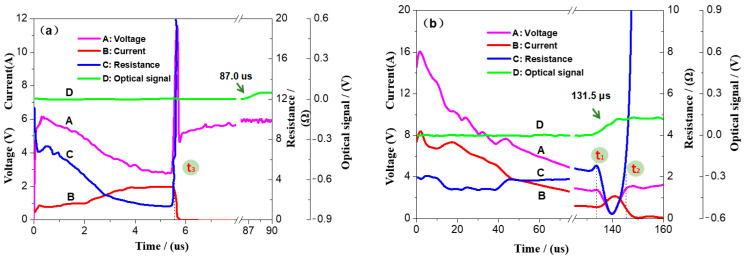
The VCRO curve of the igniting parts of the semiconductor bridge; (**a**) is the VCRO curve of D1-LS; (**b**) is the VCRO curve of D2-nAl/MoO_3_/RGO (fiber).

The capacitor discharge experiment was carried out on the above-mentioned semiconductor bridge ignition parts. By examining experimental phenomena and voltage-current-resistance-optical signal (VCRO) curves, the ignition situation of these igniting parts and the match between the micro–nano thermite charge and the semiconductor bridge igniting parts were analyzed. The results are shown in Figure 9. The charging voltage of the ignition component of the D1-LS semiconductor bridge is 5.69 V, the semiconductor bridge bursts, and the ignition component ignites. The charging voltage of the ignition component of the D2-nAl/MoO_3_/RGO (fiber) semiconductor bridge is 16.00 V, the ignition component ignites, but the semiconductor bridge does not burst. In Figure 9a, the resistance value of the ignition element of the D1-LS abruptly changes to infinity at point t_3_. At this time, the semiconductor bridge is transformed into high-heat steam or plasma after the explosion, and then the latter penetrates the charge and ignites the agent at the same time, which conforms to the characteristics of the electric explosion fire mechanism. In Figure 9b, the resistance of the D2-nAl/MoO_3_/RGO (fiber) semiconductor bridge ignition element has experienced the process of resistance increase and decrease and continues to increase to infinity. Between t_1_ and t_2_, the resistance of the ignition element decreases to a certain value, which is the resistance value of the ignition element when the polysilicon in the semiconductor bridge chip melts and is in a liquid state. However, the light signal fluctuates around t_1_, indicating that the medicament ignites and burns to the end of the igniting part. Therefore, the ignition time of the medicine is prior to t_1_, which is in line with the characteristics of the electrothermal ignition mechanism.

## 4. Conclusions

In summary, Al/MoO_3_/NCMs were fabricated via electrostatic spinning technology. The investigation on the morphology demonstrates that the spinning surface presents a concave–convex and intermittent structure due to nAl and nMoO_3_ being filled in NC, but the solvent in NC evaporates and shrinks. Al and MoO_3_ particles of the nAl/MoO_3_/NCM thermite are both nanosized, and their particle sizes are much smaller than NC; thus, the two components can be better attach to NC fibers. Thermal conductivity results show that the addition of NCM can improve the thermal conductivity of the Al/MoO_3_ thermite, and the addition of RGO affects the thermal conductivity of the thermite significantly. The energy performance results indicate that the energy performance of Al/MoO_3_/NCM thermite spinning is the best when the value of Φ is 0.90–1.00. The addition of NCM can significantly increase the burning rate of the thermite, and the addition of RGO improves its combustion rate the most. By changing the shape of the Al/MoO_3_/NCM charge and the internal composition of the charge, the sensitivity of the agent can be adjusted, and the matching performance and use performance of the electric igniter can be improved.

## Figures and Tables

**Table 1:** The thermal conductivity test results of thermite.

Samples	Thickness (mm)	Thermal Resistance (K·m^2^·W^−1^)	Thermal Conductivity (W·m^−1^·K^−1^)
mAl/MoO_3_	0.90	1.02 × 10^−5^	88
sub-mAl/MoO_3_	0.88	1.17 × 10^−5^	75
nAl/MoO_3_	0.96	1.39 × 10^−5^	69
mAl/MoO_3_/NFG	0.87	8.13 × 10^−6^	107
sub-mAl/MoO_3_/NFG	0.92	9.89 × 10^−6^	93
nAl/MoO_3_/NFG	0.97	1.20 × 10^−5^	81
mAl/MoO_3_/CNT	0.85	8.25 × 10^−6^	103
sub-mAl/MoO_3_/CNT	0.93	1.03 × 10^−5^	90
nAl/MoO_3_/CNT	1.05	1.21 × 10^−5^	87
mAl/MoO_3_/RGO	0.99	7.23 × 10^−6^	137
sub-mAl/MoO_3_/RGO	0.86	7.11 × 10^−6^	121
nAl/MoO_3_/RGO	0.90	6.29 × 10^−6^	143

**Table 2:** Al/MoO_3_ thermite spinning formula and calculation results of energy parameters under different Φ values.

Φ	Component (wt.%)				*T* (K)	*Q* (kJ·kg^−1^)	*V*_g_ (m^3^·kg^−1^)	*Mcond*	*P* (MPa)
	Al	MoO_3_	NCM	NC					
1.00	19.1	50.9	0	30.0	2983.48	3902.60	3.58	0.640	1.67
1.10	20.4	49.6	0	30.0	3055.67	3860.39	3.67	0.663	1.71
1.20	21.7	48.3	0	30.0	3170.90	3773.74	3.84	0.704	1.78
1.30	22.9	47.1	0	30.0	3194.47	3727.61	3.90	0.714	1.80
1.40	24.1	45.9	0	30.0	3186.50	3678.75	3.93	0.713	1.82
1.50	25.2	44.8	0	30.0	3152.29	3627.50	3.93	0.704	1.84
1.60	26.2	43.8	0	30.0	3099.25	3574.39	3.92	0.689	1.86
1.70	27.2	42.8	0	30.0	3032.81	3519.79	3.89	0.670	1.86
1.80	28.2	41.8	0	30.0	2955.88	3463.91	3.85	0.649	1.85
1.90	29.1	40.9	0	30.0	2895.68	3407.19	3.85	0.624	1.72

**Table 3:** Al/MoO_3_/NCM thermite spinning formula and calculation results of energy parameters under different Φ values.

Φ	Component (wt.%)				*T* (K)	*Q* (kJ·kg^−1^)	*V*_g_ (m^3^·kg^−1^)	*Mcond*	*P* (MPa)
	Al	MoO_3_	NCM	NC					
0.60	12.1	53.9	4.0	30.0	2355.39	4026.62	3.26	0.579	1.62
0.70	13.7	52.3	4.0	30.0	2528.98	3948.45	3.52	0.605	1.74
0.80	15.2	50.8	4.0	30.0	2611.14	3908.98	3.64	0.618	1.80
0.90	16.6	49.4	4.0	30.0	2761.52	3828.96	3.88	0.646	1.91
1.00	18.0	48.0	4.0	30.0	2808.27	3786.13	3.97	0.655	1.94
1.10	19.3	46.7	4.0	30.0	2740.95	3730.43	3.93	0.641	1.85
1.20	20.5	45.5	4.0	30.0	2635.50	3669.94	3.84	0.619	1.67
1.30	21.6	44.4	4.0	30.0	2452.31	3511.44	3.70	0.573	1.48
1.40	22.7	43.3	4.0	30.0	2455.12	3494.76	3.64	0.575	1.44
1.50	23.8	42.2	4.0	30.0	2458.63	3441.24	3.62	0.571	1.43

**Table 4:** Linear combustion rate of thermite.

Samples	Average Burning Rate (m·s^−1^)	Standard Deviation (m·s^−1^)
mAl/MoO_3_	4.23	1.35
sub-mAl/MoO_3_	5.56	1.14
nAl/MoO_3_	28.1	9.4
mAl/MoO_3_/NFG	26.5	10
sub-mAl/MoO_3_/NFG	39.3	7.1
nAl/MoO_3_/NFG	192	65
mAl/MoO_3_/CNT	81.6	23
sub-mAl/MoO_3_/CNT	163	51
nAl/MoO_3_/CNT	471	108
mAl/MoO_3_/RGO	155	27
sub-mAl/MoO_3_/RGO	252	76
nAl/MoO_3_/RGO	736	159

**Table 5:** Structural parameters of semiconductor bridge transducer.

Number	l (um)	w (um)	θ (º)	A (um^2^)	R_0_ (Ω)
D1	21	50.5	60	679	4.27
D2	70	380.0	60	22356	1.15

**Table 6:** The critical ignition voltage result of the semiconductor bridge.

Number	Experimental Result				Theoretical Value
	*U_cr_* (V)	*σ* (V)	*U_nf_* (V)	*U_af_* (V)	*U_tc_* (V)
D1	4.08	0.07	3.86	4.30	-
D1-N-LS	4.22	0.10	3.91	4.53	4.04
D1-nAl/MoO_3_/RGO(fiber)	4.31	0.09	4.03	4.59	3.87
D2	16.51	0.93	13.64	19.38	-
D2-N-LS/LA	11.90	1.65	6.80	17.00	13.50
D2-nAl/MoO_3_/RGO(fiber)	12.93	0.98	9.90	15.96	14.25
D2-nAl/MoO_3_/RGO(membrane)	15.02	1.31	10.97	19.07	14.13

*U_cr_* is test critical ignition voltage; *U_af_* is total ignition voltage; *U_nf_* is total non-ignition voltage; *U_tc_* is theoretical critical ignition voltage.

## Data Availability

Data are contained within the article.
